# Brief Report: Earlier Referral to Differentiated Antiretroviral Therapy Delivery at Six Months After Initiation: A Retrospective Cohort Study in KwaZulu-Natal, South Africa

**DOI:** 10.1097/QAI.0000000000003917

**Published:** 2026-09-11

**Authors:** Johan S. van der Molen, Kwena Tlhaku, Lara Lewis, Yukteshwar Sookrajh, Lungile Hobe, Thulani Ngwenya, Mlungisi Khanyile, Thokozani Khubone, Nigel Garrett, Jennifer A. Brown, Jienchi Dorward

**Affiliations:** a Centre for the AIDS Programme of Research in South Africa (CAPRISA), University of KwaZulu–Natal, Durban, South Africa;; b Discipline of Public Health Medicine, University of KwaZulu-Natal, Durban, South Africa;; c Department of Statistical Sciences, University of Cape Town, Cape Town, South Africa;; d eThekwini Municipality Health Unit, Durban, South Africa;; e Mseleni Hospital, Nhlamvu, South Africa;; f Bethesda Hospital, Ubombo, South Africa;; g Desmond Tutu HIV Centre, University of Cape Town, Cape Town, South Africa;; h Division of Clinical Epidemiology, Department of Clinical Research, University Hospital Basel, Basel, Switzerland; and; i Nuffield Department of Primary Care Health Sciences, University of Oxford, Oxford, United Kingdom.

**Keywords:** HIV, differentiated service delivery, antiretroviral therapy, retention in care, viral suppression, South Africa

## Abstract

In April 2020, South Africa adopted the World Health Organization revised eligibility for differentiated service delivery, which changed from requiring virologically suppressed clients to be on antiretroviral therapy (ART) for 12 months to 6 months. To evaluate the uptake of early community–based ART (ec-ART) and subsequent clinical outcomes, we performed a retrospective cohort study using routine, deidentified data from 124 public clinics in KwaZulu-Natal, South Africa. We included people with HIV aged ≥16 years, newly initiated on ART and virally suppressed (<50 copies/mL) at 6 months. We assessed uptake of ec-ART among people initiating ART between January 2020 and December 2022, and clinical outcomes of retention in care, death, and viremia at 12 months among those initiating ART between January 2020 and March 2022. We used multivariable Poisson regression models with robust standard errors to compare outcomes. Among 27,855 people eligible for ec-ART, 61% were women, median age was 33 years. Approximately 12.3% received ec-ART, at a median of 223 days after ART initiation. Rates of ec-ART increased from 7.0% in Q1 2020 to 20% in Q4 2022. Among 21,106 participants with outcome data, 9.5% received ec-ART. The proportion not retained-in-care/died at 12 months was 3.9% for those with and 7.9% for those without ec-ART (risk ratio 0.48; 95% confidence interval: 0.38–0.60; *P* < 0.05). Among those retained-in-care, a 12-month viral load result was available for 83.4% patients, of these 9.0% with and 10.5% without ec-ART had viremia (not significantly different). Uptake of ec-ART was low but associated with better retention and similar clinical outcomes.

## INTRODUCTION

There is growing evidence that, among clinically stable clients, using community-based antiretroviral therapy (ART) is as effective in maintaining retention-in-care and viral suppression as clinic-based care.^1–3^ Community-based ART is a differentiated service delivery (DSD) model in which clinically stable clients are referred from primary health care clinics to receive ongoing ART through community-based pickup points, adherence clubs, or other out-of-facility mechanisms, with reduced frequency of clinic visits. During the COVID-19 pandemic, the World Health Organization revised its definition of clinical stability as a criterion for referral to community-based ART. The previous definition required clients to be on ART for at least 12 months with 2 suppressed viral loads (VLs) and no current illness^4^; this was changed to at least 6 months on ART with 1 suppressed VL postinitiation and no current illness.^5^ Clients who achieved this clinical stability would be eligible for enrollment in community-based ART programs. Despite widespread adoption of community-based ART delivery, evidence on the uptake and outcomes of early referral—particularly using routine, large-scale programmatic data—remains limited.

Before the World Health Organization revised the eligibility criteria for community-based ART, we conducted a retrospective cohort study on people living with HIV on first-line ART and found that the number of clients eligible far exceeded the number referred.^3^ Qualitative findings suggested that a lack of provider buy-in to the expansion of eligibility into these programs was causing reluctance to refer clients eligible at 6 months,^6^ despite the perceived benefits by stable patients.^7^ Providers reported concerns about client readiness, fear that adherence would be worse, and the limited empirical evidence on outcomes following earlier referral. Therefore, we aim to evaluate the uptake of early referral to community-based ART programs (early community–based ART) and to compare subsequent outcomes between people referred for early community–based ART, with those not referred, to address this critical evidence gap and inform policy and implementation.

## METHODS

### Study Design and Participants

We conducted a retrospective cohort study using deidentified electronic health data from 124 public clinics in eThekwini, uMgungundlovu and uMkhanyakude districts in KwaZulu-Natal province, South Africa. We analyzed 2 cohorts: 1 to evaluate uptake of early community–based ART, and 1 to evaluate subsequent outcomes. In the uptake cohort, we included adults (≥16 years) newly initiated on tenofovir disoproxil fumarate/emtricitabine/efavirenz or tenofovir disoproxil fumarate/lamivudine/dolutegravir between 1 January 2020 and 1 December 2022, who did not have tuberculosis, were not pregnant, and were virally suppressed (VL < 50 copies/mL) 6 months after ART initiation. We excluded people who transferred-in after ART initiation because of unavailable ART initiation data, and people who became lost to follow-up before 9 months as they may not have had the opportunity to be referred for early community–based ART. For the outcome cohort, a subset of the uptake cohort, we excluded people initiated after 1 March 2022, as they did not have enough follow-up time to determine outcomes before the administrative data cut on 1 September 2023. We also excluded people who had been referred into community-based ART with a 12-month prescription,^3,6^ as they would not have been scheduled a clinic visit before the end of follow-up and so retention-in-care could not be assessed.

### Data Sources and Management

We used deidentified data from TIER.Net, an electronic health register containing demographic, clinical, and visit data for clients in South Africa's public ART program.^8^ TIER.Net provides information on clinic visits, referral for community ART delivery within the Central Chronic Medicines Dispensing and Distribution program, ART regimens, prescription durations, outcomes, and CD4 and VL measurements.

### Variables

The primary exposure was a binary variable of early community–based ART referral (defined as receiving a community ART referral to an external pickup point within 270 days of ART initiation), or no early referral. Baseline variables in TIER.Net considered as potential confounders were age, sex, ART regimen, and CD4 count at initiation.

The primary outcomes were not retained-in-care or died, and viremia at 12 months postinitiation. Clients were classified as not retained-in-care or dead at 12 months if they missed a visit by more than 90 days or died, within 9–15 months of ART initiation. Viremia was defined as a VL ≥ 50 copies/mL, using the VL closest to 12 months within a window of 9–15 months postinitiation.

### Statistical Analysis

We first described overall uptake of early community–based ART in the uptake cohort. Then, in the outcome cohort, we used univariable and multivariable regression analyses to examine associations between the primary exposure of early community–based ART referral and the outcome of not retained-in-care or died at 12 months. Among those retained-in-care with a 12-month VL, a second analysis assessed viremia at 12 months. We used generalized linear models (Poisson family) with robust standard errors, reporting risk ratios (RRs) with 95% confidence intervals (CIs). In the multivariable analyses, we adjusted for the potential confounders of age, gender, CD4 count, district, and ART regimen.

### Ethical Approval

This work was approved by the University of KwaZulu-Natal Biomedical Research Ethics Committee (BE646/17), the KwaZulu-Natal Department of Health's Provincial Health Research Ethics Committee (KZ_201807_021), and the eThekwini Municipality Health Unit, with a waiver for informed consent for analysis of anonymized, routinely collected data.

## RESULTS

### Client Characteristics

Across the 124 clinics, 70,551 ART-naïve adults initiated on ART between 1 January 2020 and 1 December 2022 and were not pregnant or diagnosed with tuberculosis. Of these, 42,696 (60.5%) were excluded as they were not on a tenofovir disoproxil fumarate/emtricitabine/efavirenz or tenofovir disoproxil fumarate/lamivudine/dolutegravir regimen (n = 392), were lost to follow-up (n = 23,623) or missed a visit by ≥90 days (n = 3441) in the firsts 9 months, did not have a VL measurement recorded at 6 months (n = 8990), or were not virally suppressed (n = 6250). Among 27,855 people eligible for early community–based ART and included in the uptake cohort, 17,111 (61%) were women (Table 1), and the median age was 33 years (IQR 27–40). Of these, 3427 (12.3%) received early community–based ART, at a median of 223 days (IQR: 199 to 242) after ART initiation. The proportion with early referral to community-based ART increased from 7.0% of those initiated in Q1 2020 to 20% of those initiated in Q4 2022 (Table 1).

**TABLE 1.** Baseline Characteristics in the Uptake Cohort

	Overall n = 27,855*	Early Community ART n = 3,427*	No or Later Community ART n = 24,428*
Gender			
Male	10,744 (39)	1257 (12)	9487 (88)
Female	17,111 (61)	2170 (13)	14,941 (87)
Age group (yr)			
16–20	1183 (4.2)	124 (10)	1059 (90)
21–30	9813 (35)	1263 (13)	8550 (87)
31–40	10,317 (37)	1242 (12)	9075 (88)
41–50	4618 (17)	577 (12)	4041 (88)
>50	1924 (6.9)	221 (11)	1703 (89)
Quarter of initiation			
Q1 2020	3717 (13)	259 (7.0)	3458 (93)
Q2 2020	2368 (8.5)	147 (6.2)	2221 (94)
Q3 2020	2324 (8.3)	213 (9.2)	2111 (91)
Q4 2020	2387 (8.6)	229 (9.6)	2158 (90)
Q1 2021	2654 (9.5)	351 (13)	2303 (87)
Q2 2021	2393 (8.6)	253 (11)	2140 (89)
Q3 2021	2023 (7.3)	258 (13)	1765 (87)
Q4 2021	2001 (7.2)	344 (17)	1657 (83)
Q1 2022	2463 (8.8)	493 (20)	1970 (80)
Q2 2022	1917 (6.9)	266 (14)	1651 (86)
Q3 2022	2197 (7.9)	338 (15)	1859 (85)
Q4 2022	1411 (5.1)	276 (20)	1135 (80)
Initiation CD4 count (cells/µL)			
≤200	4663 (17)	469 (10)	4194 (90)
201–350	5386 (19)	638 (12)	4748 (88)
351–500	5110 (18)	708 (14)	4402 (86)
>500	9150 (33)	1248 (14)	7902 (86)
Missing	3546 (13)	364 (10)	3182 (90)
ART regimen			
Tenofovir disoproxil fumarate/lamivudine/dolutegravir	23,192 (83)	3110 (13)	20,082 (87)
Tenofovir disoproxil fumarate/emtricitabine/efavirenz	4663 (17)	317 (6.8)	4346 (93)
Days to first VL	184 (169, 200)	176 (168, 195)	186 (169, 201)

* n (%); Median (Q1, Q3).

### Outcomes Retained in Care and Viral Suppression

A further 6749 people were excluded from the outcome cohort as they initiated after March 2022 (n = 6359) or received a 12-month script when starting community-based ART (meaning they would have no scheduled visits during the follow-up period with which to assess retention-in-care, n = 390). The remaining 21,106 people had initiated ART between 1 January 2020 and 1 March 2022, were eligible for early community–based ART, and were included in the outcome cohort. Of these, 1995 (9.5%) received early community–based ART and 19,111 (90.5%) did not (Table 2). The proportion not retained-in-care or died at 12 months was 77/1995 (3.9%) for those with and 1518/19,111 (7.9%) for those without early community–based ART (multivariable adjusted RR 0.48; 95% CI: 0.38–0.60; *P* < 0.05; Table 2). For the viremia analyses, among people retained-in-care, VL data were available for 16,272/19,511 (83.4%) patients, and of these 143/1592 (9.0%) with and 1546/14,680 (10.5%) without early community–based ART had viremia (RR 0.90; 95% CI: 0.76–1.06; *P* = 0.193; Table 2).

**TABLE 2.** Univariable and Multivariable RRs of Early Versus No or Later Community ART Referral and the Outcomes for Not Retained in Care or Died at 12 months and Viremia

	n/N (%)	RR Univariable	*P*	RR Multivariable	*P*
**Not retained or died at 12 months**					
Early community ART referral					
No	1518/19,111 (7.9)	1	—	—	—
Yes	77/1995 (3.9)	0.49 (0.39–0.61)	<0.05	0.48 (0.38–0.60)	<0.05
Gender					
Male	683/8130 (8.4)	1	—	—	—
Female	912/12,976 (7.0)	0.84 (0.76–0.92)	<0.05	0.76 (0.69–0.85)	<0.05
Age group (yr)					
16-20	82/880 (9.3)	1	—	—	—
21-30	660/7499 (8.8)	0.94 (0.76–1.18)	0.609	0.93 (0.75–1.16)	0.530
31-40	589/7871 (7.5)	0.80 (0.64–1.00)	0.051	0.75 (0.60–0.94)	<0.05
41-50	201/3450 (5.8)	0.63 (0.49–0.80)	<0.05	0.58 (0.45–0.74)	<0.05
>50	63/1406 (4.5)	0.48 (0.35–0.66)	<0.05	0.45 (0.33–0.62)	<0.05
Initiation CD4 count (cells/µL)					
≤200	239/3542 (6.7)	1	—	—	—
201-350	279/4161 (6.7)	0.99 (0.84–1.17)	0.941	0.97 (0.82–1.15)	0.757
351-500	253/3866 (6.5)	0.97 (0.82–1.15)	0.725	0.95 (0.80–1.13)	0.576
>500	560/6872 (8.1)	1.21 (1.04–1.40)	<0.05	1.22 (1.05–1.41)	<0.05
Missing	264/2665 (9.9)	1.47 (1.24–1.74)	<0.05	1.47 (1.24–1.74)	<0.05
ART regimen					
Tenofovir disoproxil fumarate/lamivudine/dolutegravir	1265/16,582 (7.6)	1	—	—	—
Tenofovir disoproxil fumarate/emtricitabine/efavirenz	330/4524 (7.3)	0.96 (0.85–1.07)	0.451	0.95 (0.84–1.07)	0.359
Health district					
eThekwini	952/12,933 (7.4)	1	—	—	—
Umgungundlovu	552/7023 (7.9)	1.07 (0.97–1.18)	0.202	1.12 (1.01–1.24)	<0.05
Umkhanyakude	91/1150 (7.9)	1.07 (0.87–1.32)	0.492	1.07 (0.87–1.31)	0.538
**Viremia**				
Early community ART referral					
No	1546/14,680 (10.5)	1	—	—	—
Yes	143/1592 (9.0)	0.85 (0.72–1.00)	0.056	0.90 (0.76–1.06)	0.193
Gender					
Male	815/6093 (13.4)	1	—	—	—
Female	874/10,179 (8.6)	0.64 (0.59–0.70)	<0.05	0.74 (0.68–0.82)	<0.05
Age group (yr)					
16–20	68/670 (10.1)	1	—	—	—
21–30	481/5693 (8.4)	0.83 (0.65–1.06)	0.136	0.76 (0.59–0.96)	<0.05
31–40	693/6071 (11.4)	1.12 (0.89–1.42)	0.329	0.89 (0.70–1.12)	0.320
41–50	325/2703 (12.0)	1.18 (0.93–1.52)	0.179	0.90 (0.70–1.16)	0.433
>50	122/1135 (10.7)	1.06 (0.80–1.40)	0.689	0.84 (0.63–1.11)	0.211
Initiation CD4 count (cells/µL)					
≤200	457/2835 (16.1)	1	—	—	—
201–350	383/3294 (11.6)	0.72 (0.64–0.82)	<0.05	0.76 (0.67–0.86)	<0.05
351–500	291/3040 (9.6)	0.59 (0.52–0.68)	<0.05	0.64 (0.56–0.74)	<0.05
>500	373/5252 (7.1)	0.44 (0.39–0.50)	<0.05	0.50 (0.43–0.56)	<0.05
Missing	185/1851 (10.0)	0.62 (0.53–0.73)	< 0.05	0.64 (0.54–0.75)	< 0.05
ART regimen					
Tenofovir disoproxil fumarate/lamivudine/dolutegravir	1410/12,789 (11.0)	1	—	—	—
Tenofovir disoproxil fumarate/emtricitabine/efavirenz	279/3483 (8.0)	0.73 (0.64–0.82)	<0.05	0.82 (0.72–0.93)	<0.05
Health district					
eThekwini	1138/10,173 (11.2)	1	—	—	—
Umgungundlovu	467/5203 (9.0)	0.80 (0.72–0.89)	<0.05	0.80 (0.73–0.89)	<0.05
Umkhanyakude	84/896 (9.4)	0.84 (0.68–1.03)	0.101	0.81 (0.66–1.00)	<0.05

## DISCUSSION

Using a large retrospective cohort of adults initiated on ART with viral suppression at 6 months, we assessed uptake of early community–based ART and compared clinical outcomes of clients who received early community–based ART vs those who did not. We found that among those eligible for early community–based ART, only 12% were referred. Although uptake increased from 7% in 2020 to 20% in 2022, it remained relatively low. Twelve-month retention-in-care was higher among clients who received early community–based ART compared with those who did not, and viral suppression was similar.

Although there is limited evidence specifically assessing early referral to community ART delivery programs in South Africa, our findings are consistent with existing research showing low uptake of community-based ART regardless of the timing of referral. A retrospective cohort study by our team found that the number of clients eligible for community-based ART far exceeded the number of those actually referred.^3^ We are unable to determine the reasons for this using our data; however, in a qualitative study by our team, health care workers reported that they did not think 6-month referrals were appropriate stating that although clients were virally suppressed at that point, they had not yet fully understood the treatment and struggled with adherence often forgetting their doses for several nights.^6^ Qualitative evidence from Uganda suggests potential barriers to DSD implementation to span across multiple levels—individual, health system, community, and context—where challenges such as client preferences and insufficient health system resources affect referrals.^9^

Our findings on retention-in-care are also consistent with other existing research. Evidence from a retrospective cohort study in Zambia reported very low loss to follow-up rates at 18 months among early (<6 months, 3%) and established (≥6 months, 5%) enrollers into 6 DSD models, including both facility- and community-based models. These findings suggest a strong retention overall, although significantly better among early enrollers.^10^ Various other studies have also shown that the use of various DSD models not limited to community-based ART is associated with improved retention-in-care.^11,12^ A retrospective cohort study in Mozambique evaluating the effect of three-monthly versus monthly ART dispensing on HIV care retention found that three-monthly dispensing was associated with higher retention, including among people who were enrolled early at <6 months on ART.^11^ However, a cluster-randomized clinical trial in Lesotho reported contrasting evidence, suggesting that certain client groups may still face challenges with engagement despite community-based ART.^13^

Regarding viral suppression, although our results showed similar, high viral suppression at 12 months between clients who received early community–based ART and those who did not, a pilot randomized controlled trial in Rwanda found that viral suppression rates were higher at 12 months among participants who entered DSD at 6 months with either 1 or 2 suppressed VLs, compared with those who received the standard of care, although the study was not powered for a formal comparison.^14^ Generally, various forms of community-based ART have been successful in improving viral suppression.^12^ Altogether, these findings suggest that stable ART patients can start community-based ART early without affecting viral suppression.

One of the key strengths of this study is the large dataset derived from 124 clinics across both urban and rural regions of the province of KwaZulu-Natal, enhancing the generalizability of our findings. However, a limitation of this study is the exclusion of approximately one-third of the patients because of missing VL data at the 6-month follow-up. This exclusion introduces the potential for bias, as those with missing VL data may differ in important ways from those included in the analysis, potentially skewing the study's outcomes. In addition, the possibility of unmeasured confounding, especially if clinicians selectively referred patients for early community–based ART who they anticipated would have better outcomes, cannot be ruled out. This and other factors such as the social economic status of patients in the service areas of different clinics could influence the observed results and should be considered when interpreting the findings. Finally, over 23,000 people became lost to follow-up before 6 months and so could not benefit from early community ART referral. Further research is needed to determine whether even earlier community ART referral (eg, at 3 or 4 months) would help prevent early loss to follow-up.

The findings of this study have several implications for policy and research. The low uptake of early community–based ART highlights the need for interventions targeted at improving referral rates. Policy efforts should prioritize addressing barriers affecting referrals, such as addressing health care worker concerns about client readiness and strengthening training to improve their confidence in implementing early referral to community-based ART. To support these efforts, further research is needed to better understand and quantify barriers to uptake of early community–based ART and explore ways of improving health care worker confidence and patient readiness for early referral. Future studies should also focus on ways to up-scale the implementation of early community–based ART. The South African 2023 ART clinical guidelines now support even earlier referral (<6 months on ART),^15^ and we plan to assess uptake and subsequent outcomes of this change in policy.

## CONCLUSION

In conclusion, our findings suggest that early referral to community-based ART does not result in worse outcomes. Because DSD models are generally preferred by clients and help ease the burden on clinics, early referral could be a valuable approach to improving HIV care. Although uptake remains relatively low, further work is needed to scale up early referral to community-based ART. Understanding and addressing barriers to uptake will be critical in optimizing early community–based ART and other DSD models. Refining policies and strengthening research efforts can help ensure that early community–based ART is implemented effectively for long-term benefits in HIV care outcomes.

## References

[1] Long L Kuchukhidze S Pascoe S . Retention in care and viral suppression in differentiated service delivery models for HIV treatment delivery in sub‐Saharan Africa: a rapid systematic review. J Int AIDS Soc. 2020;23:e25640. 33247517 10.1002/jia2.25640 PMC7696000

[2] Lewis L Sookrajh Y Gate K . Differentiated service delivery for people using second‐line antiretroviral therapy: clinical outcomes from a retrospective cohort study in KwaZulu‐Natal, South Africa. J Int AIDS Soc. 2021;24(suppl 6):e25802. 34713545 10.1002/jia2.25802 PMC8554220

[3] Lewis L Sookrajh Y van der Molen J . Long‐term usage patterns and clinical outcomes in a community‐based differentiated antiretroviral therapy delivery programme in South Africa. J Int AIDS Soc. 2023;26:e26141. 40698878 10.1002/jia2.26141 PMC10354003

[4] World Health Organization. Consolidated Guidelines on the Use of Antiretroviral Drugs for Treating and Preventing HIV Infection. Geneva, Switzerland: WHO; 2016. Available at: https://www.who.int/publications/i/item/9789241549684. 27466667

[5] World Health Organization. Updated Recommendations on Service Delivery for the Treatment and Care of People Living with HIV. World Health Organization; 2021. 33999550

[6] Tlhaku K Msimango L Sookrajh Y . Healthcare worker perspectives on adaptations to differentiated anti-retroviral therapy delivery during COVID-19 in South Africa: a qualitative inquiry. PLoS Glob Public Health. 2024;4:e0003517. 39121062 10.1371/journal.pgph.0003517 PMC11315304

[7] Pascoe SJ Scott NA Fong RM . “Patients are not the same, so we cannot treat them the same”–A qualitative content analysis of provider, patient and implementer perspectives on differentiated service delivery models for HIV treatment in South Africa. J Int AIDS Soc. 2020;23:e25544. 32585077 10.1002/jia2.25544 PMC7316408

[8] Osler M Hilderbrand K Hennessey C . A three‐tier framework for monitoring antiretroviral therapy in high HIV burden settings. J Int AIDS Soc. 2014;17:18908. 24780511 10.7448/IAS.17.1.18908 PMC4005043

[9] Zakumumpa H Rujumba J Kwiringira J . Understanding implementation barriers in the national scale-up of differentiated ART delivery in Uganda. BMC Health Serv Res. 2020;20:222. 32183796 10.1186/s12913-020-5069-y PMC7077133

[10] Jamieson L Rosen S Phiri B . How soon should patients be eligible for differentiated service delivery models for antiretroviral treatment? Evidence from a retrospective cohort study in Zambia. BMJ Open. 2022;12:e064070. 10.1136/bmjopen-2022-064070 PMC9772670 36549722

[11] Saura-Lázaro A Augusto O Fernández-Luis S . HIV care retention in three multi-month ART dispensing: a retrospective cohort study in Mozambique. AIDS. 2024;38:1402–1411. 38652496 10.1097/QAD.0000000000003913 PMC11216376

[12] Barnabas RV Szpiro AA van Rooyen H . Community-based antiretroviral therapy versus standard clinic-based services for HIV in South Africa and Uganda (DO ART): a randomised trial. Lancet Glob Health. 2020;8:e1305–e1315. 32971053 10.1016/S2214-109X(20)30313-2 PMC7527697

[13] Amstutz A Lejone TI Khesa L . Offering ART refill through community health workers versus clinic-based follow-up after home-based same-day ART initiation in rural Lesotho: the VIBRA cluster-randomized clinical trial. PLoS Med. 2021;18:e1003839. 34673765 10.1371/journal.pmed.1003839 PMC8568187

[14] Ross J Anastos K Hill S . Reducing time to differentiated service delivery for newly-diagnosed people living with HIV in Kigali, Rwanda: a pilot, unblinded, randomized controlled trial. BMC Health Serv Res. 2024;24:555. 38693537 10.1186/s12913-024-10950-z PMC11062003

[15] Republic of South Africa National Department of Health. 2023 ART Clinical Guidelines for the Management of HIV in Adults, Pregnancy and Breastfeeding, Adolescents, Children, Infants and Neonates. Pretoria: Republic of South Africa National Department of Health, editor. 2023.

